# The relationship between Chinese university students’ learning preparation and learning achievement within the EFL blended teaching context in COVID-19 post-epidemic era: The mediating effect of learning methods

**DOI:** 10.1371/journal.pone.0280919

**Published:** 2023-01-24

**Authors:** Meng Hua, Lin Wang

**Affiliations:** 1 International Office, Xuzhou Kindergarten Teachers College, Xuzhou, Jiangsu, China; 2 School of Preschool and Special Education, Xuzhou Kindergarten Teachers College, Xuzhou, Jiangsu, China; 3 Graduate School, Lyceum of the Philippines University, Trias City, Cavite, Philippines; Chulalongkorn University, THAILAND

## Abstract

The effectiveness of the blended teaching model in improving university students’ English learning achievement has been frequently reported in China in the post-pandemic era. However, such research has seldom explored the students’ entire EFL (English as a foreign language) learning process and mechanism from the perspective of learners within this model. This study therefore used the 3P (presage, process and product) teaching and learning theory to explore the mediating role of learning methods (i.e., learning engagement and academic procrastination) in the relationship between learning preparation (i.e., academic self-concept and course experience) and learning achievement within the Chinese EFL blended teaching context from the perspective of learners. In this study, 942 Chinese university students (male: *N* = 447; female: *N* = 495) participated in a survey and completed electronic questionnaires on EFL-related academic self-concept, learning engagement, academic procrastination, and learning achievement. The data were analyzed using AMOS software and a structural equation modeling (SEM) technique. The results showed that both students’ academic self-concept and course experience directly and positively predicted their English learning achievement. Moreover, students’ academic self-concept of learning achievement was partially mediated by learning engagement and academic procrastination, whereas the effect of course experience on learning achievement was fully mediated by learning engagement and academic procrastination. After discussing these findings, suggestions as well as limitations for future studies will be given.

## Introduction

Since 2020, traditional teaching has been hindered by COVID-19 and the rise of online teaching has ushered in a new normal for student development. Benefiting from the deep integration of information technology, online teaching has freed itself from the constraints of time and space, which can easily offer students the opportunity to learn anywhere, at any time, by any means of new media [1]. As the epidemic has been brought under control, and thus, traditional teaching has resumed, it is of great necessity to integrate the benefits of web-based online teaching approaches into traditional teaching patterns to better promote educational innovation and reform in the post-pandemic era. In the blended teaching model, 30–70% of the instruction is delivered online while the rest is delivered in a face-to-face fashion [2]. According to the data from Chinese colleges and universities, the blended teaching approach has been implemented universally [3], and satisfactory results have been achieved in the effects on university students’ learning through this method [4].

### The EFL blended teaching model and the 3P teaching and learning theory

#### The blended teaching model

Blended teaching was first introduced in 1969 as a basic constituent of the learning system of distance teaching instruction [5]. The blended learning model integrates contact teaching with instructors and self-controlled preparation using online resources [6]. In doing so, it brings together the traits of online and offline learning, including instructional modalities, teaching methods, and learning tools [7]. It is believed that self-controlled preparation of pre-class learning materials, such as previewing of book chapters, journal papers, and related videos in advance, can save much time for covering important content in class and promote more meaningful interaction between teachers and students within this model [8]. The blended model has been shown to relate to a higher level of learning and promotion of active rather than passive learning [9–11], and it has greater potential for improving students’ learning performance compared with the traditional teaching model [12,13]. Overall, the blended teaching model proves more effective than traditional teaching because it combines the benefits of both online and traditional teaching methods, which reinforces and optimizes the learning process to improve learning quality and academic achievement [12,14].

#### The EFL blended teaching model

It is commonly acknowledged that technology plays a key role in language learning [15–18]. Past research has shown that a technology-rich teaching context provides learners with the best language practice environment, which contributes to higher language performance [19,20]. According to Dousti’s research, technology-enhanced instructions provide a wealth of resources to improve students’ familiarity with the vocabulary, syntactic structure, phrases, and flow and connection of ideas in language teaching [21]. Moreover, use of technology in university foreign language classes can simplify culturally responsive teaching, enabling teachers to easily educate culturally and linguistically different language learners [22]. Therefore, within today’s digital context, university English classes have increasingly adopted the blended teaching model for the benefits of technology-supported instruction.

The blended language teaching model entails a language curriculum that integrates face-to-face classroom teaching with appropriate adoption of technology [23]. The application of the blended model is generally believed to leverage and supplement traditional instruction to increase students’ diverse skills of EFL (English as foreign language) [21]. Previous researchers have illustrated the intrinsic benefits of the blended learning method in teaching EFL, such as positive outcomes of using the mixed method on learners’ achievement, positive participation, and feelings of motivation from using the online learning system [24–27].

#### The EFL blended teaching model in China

For decades, the most important foreign language in China has been English. University graduates’ proficiency in English serves as an important factor in securing employment and pursuing future career development [28,29]. Thus, EFL courses have always been among the foundational and most essential courses in higher education in China [30]. Nowadays, as the traditional teaching model has been significantly affected by the epidemic, the blended teaching model has become the typical way in which instruction is provided in university English classes in China [31]. Under the traditional English teaching model, due to time and location constraints, Chinese university students with non-English-speaking backgrounds often have difficultly developing global literacy in lingua franca English [32]. After implementing the blended teaching model, English class is no longer a place of passive vocabulary memorizing and sentence practicing, but an immersive and participatory learning environment where university students who speak EFL can easily learn [33]. Moreover, blended teaching can enable learners to participate in more interesting learning opportunities, activate their prior knowledge, and expose them to more multimodal materials and real discourses, such as charts, websites, texts, videos, and pictures, which can accelerate the improvement of English learning [21]. Several researchers have demonstrated that compared to the traditional teaching model, the blended model has positive effects on both learning autonomy and motivation in Chinese English classes [34,35]. However, previous research on the blended foreign language teaching model has mainly discussed the influence of this model on various aspects of English learning (such as grammar, writing, and reading) from the perspective of teaching [5,20,36]. Seldom has research explored the full scope of the students’ English learning process and mechanism under the blended model from the perspective of learners. Therefore, given the importance of English learning and the prevalence of the blended teaching model during the pandemic in China, it is necessary to understand the students’ EFL learning process within the blended teaching model in the context of Chinese universities. The 3P teaching and learning theory serves as a descriptive framework in which students’ learning process can be clearly captured in a particular teaching context. Thus, this theory can provide useful guidance for understanding university students’ English learning process under the blended teaching model in China.

#### The 3P teaching and learning theory

The 3P teaching and learning theory, presented by Janette B. Biggs, provides a theoretical framework that describes how instructors’ prior teaching experiences and students’ prior learning experiences connect and link to each other to account for students’ later learning achievement [37,38]. It has been popularly applied in an effort to comprehend the learning process of students from different social and cultural backgrounds [39,40], in different educational phases including secondary education and higher education [41,42], and in different courses, including accounting, management, health and social care, and mathematics [43–46]. In the 3P teaching and learning model, the learning process of students is conceptualized into three correlative phase sequences—namely, presage, process, and product.

The *presage* phase involves individual learning preparation, which represents students’ prior learning states, as well as the external preparation by teachers, which represents teachers’ prior experiences and personalities. Individual preparation factors in the presage phase are defined as including prior experience and beliefs that students bring into the learning experience and their expectations of the new learning experience [47]. These factors refer to students’ characteristics, which include prior abilities and knowledge, preferred ways of learning, values and expectations, and interests, which are relatively stable in learning [48]. Teachers’ prior experiences and personalities refer to teachers’ professional academic skills, expertise, teaching style, course design ability, and classroom climate [44,49]. Both the internal and external learning preparation variables exist prior to classroom learning [50,51].

The *process* phase emphasizes how students develop diverse learning methods after class teaching occurs. This phase systematically describes the changes in students’ self-directed learning or passive learning after the interaction between individual and external learning preparation factors [49]. During this period, depending on how confident students feel in their ability to master the learned knowledge and how difficult, important, and enjoyable the tasks are, students will develop different learning motivations, leading them to choose different learning strategies and particular learning methods [45].

Finally, the *product* phase refers to the learning outcome, including the performance and achievement of students after learning. The outcomes of learning have a vital influence on later learning motivation and involvement [38]. By analyzing the outcome, students can evaluate the appropriateness of their learning strategies and readjust their learning efforts as needed. Teachers can also examine their teaching effectiveness according to students’ learning outcomes and change their teaching methods accordingly [49].

The three phases proposed in the 3P theoretical model are intertwined and form a dynamic learning system as a whole [52]. More specifically, certain aspects of the individual preparation factors, as well as the external teaching preparation factors, will facilitate metacognitive activity related to students’ awareness of their own learning motivation and will control the learning methods used by the students to complete a certain learning task [37]. Then, learning methods will further influence students’ learning achievement. That is to say, learning methods play a mediating role between learning preparation and learning achievement. Meanwhile, variables relating to learning preparation may also have a direct effect on learning achievement. The 3P theory provides us with a cycle of events from the presage phase to process phase and product phase, which helps us to understand the entire scope of university students’ learning process.

The choice of Biggs’ 3P theory as the framework for this research not only because it is helpful to explore the learning process from the perspective of students, but also because it is consistent with the blended teaching model in several respects. First, the 3P theoretical model adopts a constructivist perspective because it regards learners as self-determining subjects who actively choose information from the perceived environment, and who construct new knowledge based on what they already know [53]. This aligns with the nature of the blended teaching model, which emphasizes how learners can build their own understanding and knowledge through action and reflection in interactive classes [54]. Second, the roles of students and teachers are the same in both Biggs’ 3P theory and the blended teaching model. Both highlight the importance of learners’ personal learning quality and learning style, including their autonomy, motivation, and self-efficacy [5,14]. Importantly, their learning depends on the student characteristics described above in the discussion of the 3P model. The blended teaching model effectively activates the previously mentioned elements of students’ characteristics [55]. Teachers should assume not only a leading role in transmitting knowledge, but also in guiding, enlightening, and monitoring the teaching process, according to both Biggs’ 3P theory and the blended teaching model [23,37]. In short, they both attach importance to the organic combination of the teacher’s leading position and the student’s central role. Third, they both advocate for the importance of teaching context. In the 3P model, a diversified and positive teaching context encompasses all factors under the teacher’s control, all of which have significant motivational consequences in the next two stages [37], while the adoption of blended teaching in university EFL classes can provide students with an immersive, supportive, constructive, and highly participatory teaching context [33,37]. Therefore, it is reasonable to understand the key psychological process of university students’ EFL learning based on the framework of the 3P theory. The current study explores the mechanism and process of learning employed by students in EFL classes using the 3P model within the Chinese university context, examining the relationship among learning preparation, learning methods, and learning achievement. This is a worthwhile topic for investigation because of the current educational needs created by the global epidemic.

### Overview of the hypotheses development

**Fig 1:**
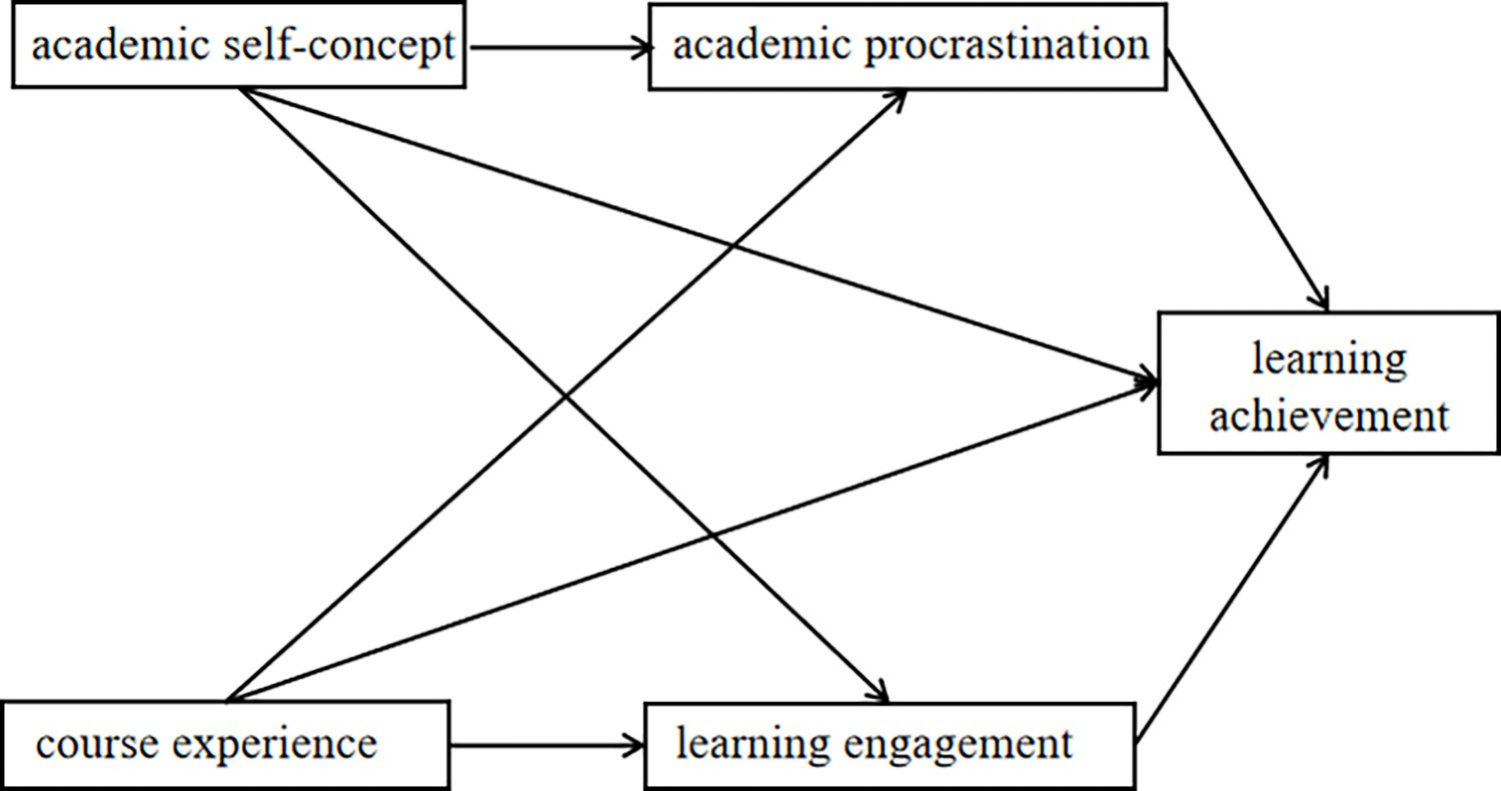
Mediation model of university students’ EFL learning process within blended teaching model context.

This study is the first to simultaneously examine the structural relationships between presage, process, and product in the Chinese EFL learning environment within the blended teaching context with the help of the 3P teaching and learning theory. To better understand this theoretical model (Fig 1), the various paths demonstrating the relationships of specific typical variables in the three phases are explained in the following section.

#### The path from learning preparation to learning achievement

According to the 3P teaching and learning theoretical model, learning preparation in the presage phase consists of individual learning preparation and external teaching preparation. The former refers to the students’ learning characteristics, including their prior knowledge of the subject they are learning about, their interest in it, their learning ability, and other factors [37,51]. Among these qualities, academic self-concept serves as a very important factor for individuals, reflecting students’ prior experiences, personalities, abilities, beliefs, and other learning characteristics in the presage phase because it involves their degree of accumulation of knowledge and self-evaluation regarding a specific academic domain [56]. Academic self-concept involves the student’s self-awareness of learning ability in a learning context as well as a relatively stable view developed by the student of his or her learning ability [57]. Learning achievement, as a representative learning outcome in the product phase, reflects the quality of the university, and test scores act as the direct reflection of learning achievement. Results from empirical research on the connection between academic self-concept and learning achievement are mixed. Some researchers have found that a strong self-concept promotes high academic achievement [58,59]. For example, Marsh’s longitudinal study showed that prior academic self-concept serves as a significant factor influencing students’ subsequent standardized test scores [60]. Others have argued that a reciprocal and mutually reinforcing relationship exists between academic achievement and self-concept, with both acting as cause and effect [61,62]. Based on the previous meta-analysis studies, a student’s academic self-concept is positively and significantly associated with the learning achievement of the student in different learning phases, with the correlation coefficient stabilizing between 0.2 and 0.27 [63]. However, Huat held that once background factors or prior attainment variables are controlled, connections between academic self-concept and achievement outcomes tend to disappear [64]. It is believed that academic self-concept related to language may influence English learning in various ways; self-concept related to pronunciation, for instance, can reduce students’ anxiety in Chinese EFL classes and increase their confidence in learning English [65], so it seems that academic self-concept affects subsequent learning outcomes obviously and positively within the Chinese EFL context. Therefore, academic self-concept is very likely to directly predict learning achievement in Chinese university students’ EFL learning.

External teaching preparation in the presage phase mainly involves all factors relating to the teaching situation—for example, teachers’ teaching content, methods of teaching and assessment, teachers’ professional skills, and classroom and school climate or spiritual disposition [38]. Since course experience can describe students’ perceptions of teaching quality, evaluation, workload, and learning community, students’ view of external teaching preparation in the presage phase can be concentrated on this variable [66–68]. Previous literature has shown that course experience of college students is positively and significantly associated with their academic outcomes [69–71]. According to the findings of Guo Jianpeng’s study, when a university was equipped with a high level of teaching quality, clear teaching goals, a relatively high degree of learning freedom, a moderate amount of coursework, and reasonable assessment methods, the students realized better academic achievement [72]. The Course Experience Questionnaire (CEQ) is one of the most widely used scales for evaluating the learning experience, and it also serves as a performance indicator of the teaching effect of the whole curriculum in higher education [67,73]. The theoretical basis of the CEQ is that students’ perceptions of curriculum, teaching, and assessment standards act as key determinants of the quality of their learning methods and outcomes [74]. In many previous studies, university students’ examination results significantly and positively correlated with the dimensions “good teaching,” “appropriate workload,” and “appropriate assessment” in CEQ [67,68,71]. In general, in the environment of the blended teaching model, students’ English course experience was relatively good and they felt a higher sense of immersion and internal motivation [75,76]. For instance, blended teaching maximized the opportunity for learners to practice English listening using online resources freely at their convenience [55]. Moreover, the blended teaching model involved more extensive learning methods and channels than traditional learning, which proved more conducive to the development of students’ English skills and could arouse and activate positive emotions in learning preparation [77]. From this vantage point, it seems that course experience not only predicts later learning outcomes but also closely relates to the previously mentioned individual qualities of students within the EFL blended teaching context. Therefore, university students’ course experience is very likely to directly predict learning achievement within the EFL blended teaching context.

*The path from learning methods to learning achievement*. Based on the 3P teaching and learning theoretical model, students will choose different learning methods to study within the process stage after learning preparation occurs. Learning engagement is a positive learning method that centers on the degree of engagement and endeavor devoted by students to learning [78]. It includes three dimensions: vigor, dedication, and absorption. Vigor refers to the degree to which a person is willing to work hard and persevere in the face of difficulties, dedication refers to a person’s strong sense of responsibility and achievement in learning, and absorption refers to the ability of individuals to concentrate on learning for a long time and have a positive psychological experience in the learning process [79]. In addition, learning engagement includes three component parts—namely, behavioral engagement, cognitive engagement, and emotional engagement. The three components of engagement might have different relationships with academic achievement, researchers have found [80]. Although emotional, cognitive, and behavioral engagement have all been favorably correlated with academic achievement [81,82], behavioral engagement may have a stronger positive correlation with academic achievement than emotional and cognitive engagement [83]. As elucidated by the learning engagement theory, learning engagement serves as a carrier and intermediary for cognitive and emotional engagement [84]. Numerous studies have shown that for college students, learning engagement is closely linked to academic achievement, ability development, and learning satisfaction [85–87]. In studies of higher education, learning engagement often acts as a strong predictor for academic development [88]. The concept of learning engagement has grown extremely popular in higher education and is usually deemed to be on par with academic success and effective learning [89], perhaps because deeper engagement in learning can lead students to beneficial educational practices that further lead to well-rounded learning [90,91]. Considering the significance of learning engagement in any teaching environment, including face-to-face, online, and blended curriculum [92], and the well-documented positive relationship between learning engagement and learning achievement in various courses and teaching formats [93], university students’ learning engagement within the process phase is very likely to positively predict learning achievement in the product phase within the Chinese EFL blended teaching context.

Procrastination involves an irrational tendency to delay the commencement or completion of a planned behavior, or to delay making a decision [94]. It can happen in school, work, daily activities, family life, and other social interactions. Although it occurs in a variety of fields, procrastination occurs most often in activities related to learning [95]. Academic procrastination is a negative learning method applied by students in the process phase, entailing the non-rational behavior of deferring the start of the learning task or extending the deadline of the task [96]. Procrastination is the opposite of learning engagement; the two represent both extremities of the learning state in the process phase. Academic procrastination usually manifests after class, such as when completing homework, previewing the next lesson, and studying for a test [97]. Studies have shown that academic procrastination is a common and widely experienced problematic behavior among university students—95% of university students may deliberately delay their learning, and 70% may often engage in the behavior of delaying a learning task [98]. Researchers tend to regard academic procrastination as a result of self-regulation failure and consider that individuals using this negative learning approach will have negative psychological experiences such as fatigue, guilt, depression, and anxiety, which can severely affect university students’ physical and mental state [99–101]. Moreover, considerable empirical research has pointed out that using this negative learning method may lead to poor academic performance and learning achievement [102–104]. This occurs because procrastinators are so busy dealing with anxiety that they put off the task at hand until they do not have enough time to complete it, or because procrastinators do not invest the time and effort needed for performing well due to underestimating how long a particular task will take [105]. Therefore, university students’ academic procrastination during the process phase is very likely to negatively predict learning achievement in the product phase.

#### The path from learning preparation to learning methods, and then to learning achievements

On the path from learning preparation to learning methods, the relationship between individual preparation and different types of learning methods has been confirmed to a certain extent in some previous studies. In terms of the relationship between individual preparation and the negative learning method of procrastination, it is believed that the more positive the individual learning preparation is, the less procrastination students will engage in during the learning process [106]. Focusing on the relationship between academic self-concept and academic procrastination, Luo Yun et al. discussed the mechanism by which learning self-concept influences academic procrastination. In his view, a lower level of learning self-concept in students leads to a distant and indifferent attitude toward learning, more fatigue and discomfort, and a lower sense of achievement, making students more prone to procrastinate [107]. Meanwhile, in terms of the relationship between individual preparation and positive learning methods such as learning engagement, researchers have shown that students are more likely to display more learning engagement if they have a higher academic self-concept [108,109].

In addition, numerous studies have explored the relationship between external types of teaching preparation such as course experience and different types of learning methods. On one hand, Yin et al. demonstrated the connection between course experience and learning engagement. They pointed out that students’ understanding of clear teaching goals, the development of teachers’ generic skills, and an appropriate course load were positively and significantly correlated with learning engagement [42]. Coates took a more inclusive and holistic view of the students’ experience, arguing that learning engagement develops from the dynamic association between individual learning preparation and external teaching preparation [90]. On the other hand, many researchers have found that a negative relationship exists between the deep cognitive experience of a specific course and procrastination among university students [110,111].

However, previous research has mainly concentrated on the exploration of the relationship between the learning preparation variables and the learning method variables, while little research has included the learning achievement variables in the discussion and explored the relationship among the three [112–115]. Recently, more researchers have been realizing that learning methods, as process variables, would be affected by variables relating to learning preparation, which would have a sustained influence on learning achievement variables [116–118]. For example, Barattucci et al. demonstrated the mediating function of learning methods between the perceptions of the academic environment (one of the representative variables of learning preparation) and learning achievement by using the 3P teaching and learning model [119]. University students’ academic self-concept and course experience, which both represent the learning preparation variables, are therefore very likely to indirectly predict learning achievement through different learning methods, according to their findings. In addition, the 3P model has not been used to fully grasp the learning process of university students, because previous literature studying the learning mechanism with the 3P model only incorporated the influence of positive learning methods on learning achievement while failing to discuss the influencing mechanism of negative learning methods on the relationship between learning preparation and learning achievement [72,114,118]. According to the above analysis of the relationship between negative learning methods and learning preparation variables, university students’ academic self-concept and course experience are believed to negatively predict their adoption of negative learning methods—namely, academic procrastination—which in turn negatively affects their final academic achievement. In sum, we predict that within the field of EFL, university students’ academic self-concept and course experience may influence their academic achievement not only through learning engagement that represents the use of a positive learning method, but also through academic procrastination that represents negative learning methods within the blended teaching context.

### The present study and hypotheses

Taking the 3P teaching and learning theory perspective, the present study explored the mediating mechanism of students’ learning methods between learning preparation and learning achievement within the Chinese university EFL context. We proposed a multiple mediation model to investigate this concept (see Fig 1). This model is based on the previously discussed literature that encompasses how university students’ internal and external English learning preparations relate to their choice of learning methods and in turn affect their learning achievement in EFL classes. In this model, internal learning preparation refers to academic self-concept, while external study preparation refers to course experience. Positive learning method refers to learning engagement, while negative learning style refers to academic procrastination. The following hypotheses will be tested with a sample of Chinese university students in EFL classes implementing the blended teaching model.

H1a. Academic self-concept can directly predict learning achievement in Chinese university students’ EFL learning.
H1b. Course experience can directly predict learning achievement in Chinese university students’ EFL learning.
H2. The effect of academic self-concept on learning achievement is mediated by learning engagement in Chinese university students’ EFL learning.
H3. The effect of course experience on learning achievement is mediated by learning engagement in Chinese university students’ EFL learning.
H4. The effect of academic self-concept on learning achievement is mediated by academic procrastination in Chinese university students’ EFL learning.
H5. The effect of course experience on learning achievement is mediated by academic procrastination in Chinese university students’ EFL learning.

## Methods

### Participants and procedure

#### Participants

The participants were recruited from two universities located in Xuzhou City, a second-tier city in the Chinese setting. All the samples enrolled in a compulsory course named “college English”. Questionnaires were distributed to 979 first-year non-English major students, and 37 invalid questionnaires were eliminated because of their incompleteness. Finally, 942 valid questionnaires were collected, giving a response rate of 96.2%. Among the respondents, there were 447 male and 495 female students with a mean age of 18.66±.71 years old, which showed a relatively balanced gender proportion.

#### Ethics statement

The present study involving human participants were reviewed and approved by the Human Research Ethics Committee of the selected University. Before conducting the survey, participants and the English course teachers provided their written informed consent to take part in this research. Only the data of participants who agreed to participate in the questionnaire survey would be used. University students’ participants in this questionnaire was entirely voluntary and they could stop and withdraw from this survey at any time.

#### Context and procedure

The English course adopted the blended teaching model, with a frequency of 3 sessions per week. The final examination was administered at the end of the first semester, which was the only means to grade the students. Students were recruited using “Wenjuanxing”, an online crowdsourcing platform to complete the questionnaire two weeks after the final examination. They were told that they could opt-out of the questionnaire if they did not wish to attend. Even if participants refuseed to participate in the questionnaire, it would not affect them in the future. Responses were confidential and anonymous. The questionnaire took the participants about 25 minutes.

### Measures

#### EFL Academic Self-Concept (EFL-ASC)

An adapted version of the English Self Concept-Chinese Students (ESC-CS), consisting of 26 items measuring the five factors of general EFL Academic Self-Concept (6 items), listening (5 items), writing (5 items), speaking (5 items), and reading (5 items), was used according to the characteristics of Chinese students” English learning [120]. The scale was answered on a 5-point Likert-type scale (ranging from “strongly disagree” to “strongly agree”) and a total of 14 questions were designed on the EFL-ASC for reverse scoring. The higher the scale’s score, the stronger the academic self-concept of students in EFL learning. Besides, the confirmatory factor analysis showed the scale had a good model fit: *χ^2^*/df = 2.336, CFI = .998, GFI = .995, RMSEA = .038, SRMR = .0081, and the Cronbach’s α coefficient for the EFL-ASC was 0.958.

#### EFL Course Experience Questionnaire (EFL-CEQ)

An adapted version of the Course Experience Questionnaire (CEQ)[121], measuring the four factors of good teaching, generic skills, clear goals and standard, and overall satisfaction item, was used. A total of 2 questions were designed on the EFL-CEQ for reverse scoring. Based on the CEQ, the content relating to the EFL course was added to the EFL-CEQ. For example, the original question “*Overall*, *I am satisfied with the quality of the course*” was rephrased as “*Overall*, *I am satisfied with the quality of the English course*”. The scale was answered on a 5-point Likert-type scale (ranging from “strongly disagree” to “strongly agree”). The higher the questionnaire’s score, the better the experience of students in EFL learning. In addition, the model fit of EFL-CEQ was good as *χ^2^*/df = 4.18, CFI = .997, GFI = .996, RMSEA = .058 SRMR = .0091, and the Cronbach’s α coefficient for the scale was .960.

#### EFL Study Engagement Scale (EFL-SESS)

An adapted version of the Utrecht Work Engagement Scale-student (UWES-S), consisting of 14 items measuring the three factors of vigor (5 items), dedication (5 items), and absorption (4 items), was used to test students’ learning engagement [122]. The Cronbach”s α coefficient for EFL-SESS was 0.969. The scale was answered on a 7-point Likert-type scale (ranging from “strongly disagree” to “strongly agree”). On the basis of the UWES-S, each item of EFL-SESS was slighted revised to be contextualized. For example, the question “*I can persist in studying for a long time*” was revised to “*I can persist in studying English for a long time*”. The higher the scale”s score, the more engagement students have invested in English learning. In addition, the confirmatory factor analysis showed that the number of observed variables was equal to the number of parameters for this scale, so that the model had zero degrees of freedom. This kind of model is called saturated model and its model fit is perfect.

#### EFL Academic Procrastination Scale (EFL-APS)

The EFL academic procrastination of students was measured by means of an adapted version of the Short General Procrastination Scale (SGPS) [123]. It included 3 items that were answered on a 5-point Likert-type scale (ranging from “strongly disagree” to “strongly agree”). The Cronbach’s α coefficient for EFL-APS was .777. On the basis of the SGPS, each item of EFL-APS was slighted revised to be contextualized. For example, the question “*For the homework that must be done*, *I will also postpone it for a few days*.” was revised to “*For the English homework that must be done*, *I will also postpone it for a few days*.” The higher the EFL-APS’s score, the higher procrastination students have performed in EFL studies. In addition, the confirmatory factor analysis showed that the number of observed variables was equal to the number of parameters for this scale, so that the model had zero degrees of freedom. This kind of model is called saturated model and its model fit is perfect.

#### EFL learning scores

In this study, learning achievement refers to EFL course examination scores. The scores were expressed by the mean score of the 2020–2021 English examination and acquired by self-administrated questionnaires. The English examination lasted for a two-hour examination, and students had to complete four types of English questions. The examination was assessed by EFL course teachers, such that Writing contributed 20%, Words and Phrases 20%, Reading 40% and Translation 20% of the total grade. The students were only given a total grade score (this is also the only available grade score for the present research), ranging from 0 to 100. The full score of English examination is 100 points and the mean score was 74.149, with a standard deviation of 13.961. Unlike the other four measures posited as latent variables, learning achievement was treated as an observed variable in SEM analysis.

### Data statistical analysis

The hypothesized research model was a multiple mediation model in which the effect of academic self-concept and course experience on learning achievement was mediated either by learning engagement or academic procrastination. In order to analyze this model, Harman’ s single-factor test used by SPSS 22.0 and the Confirmatory Factor Analysis (CFA) used by AMOS 24 were both conducted to assess the common method variance. Then bivariate correlational tests, reliability tests and descriptive statistics analysis were conducted using SPSS 22.0. Afterwards, a structural equation model was constructed and the bias-corrected percentile bootstrap method was used to analyze the mediator effect. The said method can be applied to explore various mediating effect models with large, medium, or small samples, and estimate more accurate confidence intervals for the mediating effect size [124].

## Results

### Test of common method bias

Before the formal data analysis, Harman’s single-factor test was conducted to assess common method bias [125]. All observed variables in the present study were loaded into an exploratory factor analysis to ascertain whether the first factor would account for a majority of the variance among the variables [126]. The result indicated that the first extracted factor explained 38.707% of the variance (less than 50%), which meant that common method bias was not serious [127].

In addition, the Confirmatory Factor Analysis (CFA) was also used to estimate the common method variance. All dimensions of 4 latent variables (academic self-concept, course experience, and learning engagement, academic procrastination) and 1 significant variables (learning achievement) were included into one-factor and five-factor confirmatory factor analysis. Then, the goodness of fit indices of the one-factor model was compared with the five-factor model. The results demonstrated that the difference between the five-factor model (χ2 = 390.3, df = 96) and the one-factor model (*χ*^2^ = 244374.3, df = 136) was significant, △*χ*^2^ = 243984, △df = 40, *p*<0.001, which provided support for the fact that the common method variance would not affect the standardized path coefficients and the structural model fit indices [128,129].

### Descriptive statistics and correlation analysis

Table 1 shows the results of descriptive statistics and correlation analysis on the variables of academic self-concept, course experience, learning engagement, academic procrastination, and learning achievement. The results of the correlation analysis showed that there was a significantly positive correlation between any two variables among academic self-concept, English course experience, learning engagement, and learning achievement; and there was a significantly negative correlation between academic procrastination and other four variables.

**Table 1:** Correlation analysis between descriptive statistical results and variables.

	M	SD	1	2	3	4	5
1 academic self-concept	4.948	1.182	1				
2 course experience	3.705	.659	.433**	1			
3learning engagement	4.048	1.182	.694**	.636**	1		
4 academic procrastination	2.456	.789	-.309**	-.290**	-.274**	1	
5 learning achievement	74.149	13.961	.518**	.399**	.531**	-.284**	1

Note: ***p < .001, **p < .01, *p < .05.

### Structural model analysis

Regression analysis was used to verify Hypothesis 1 by testing the relationship between learning preparation variables (academic self-concept and course experience) and learning achievement without considering the intermediate variables learning engagement and academic procrastination. The results showed that academic self-concept had a positive direct effect on learning achievement (*β* = .435, p<0.001) and course experience had a positive direct effect on learning achievement (*β* = .211 p<0.001), which verified H1a and H1b, that is, academic self-concept and course experience can directly predict learning achievement in Chinese university students’ EFL learning.

**Fig 2:**
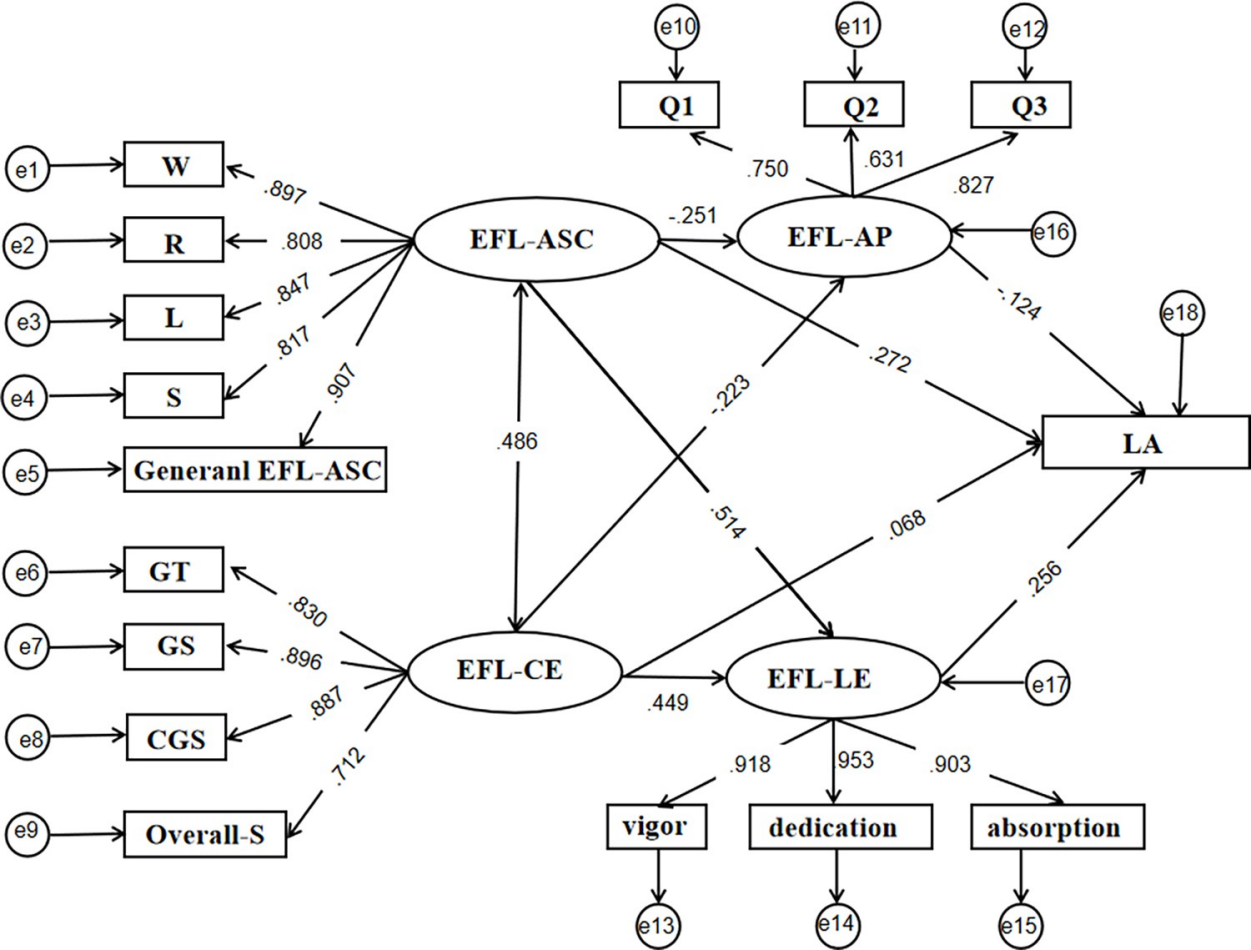
Mediation model effect plot. Note:W = Writing; R = Reading; L = Listening; S = Speaking; General EFL-ASC = General EFL Academic Self-Concept; GT = Good Teaching; GS = Generic Skills; CGS = Clear Goals and Standard; Overall-S = Overall Satisfaction; Q1 = Question 1; Q2 = Question 2; Q3 = Question 3; EFL-ASC = EFL-Academic Self-Concept; EFL-CE = EFL-Course Experience; EFL-AP = EFL-Academic Procrastination; EFL-LE = EFL-Learning Engagement; LA = Learning Achievement.

The proposed hypothetical model was tested by using the latent variable structural equation modeling. Results showed that the model had good fit indices, i.e., *χ^2^*/df = 4.068, CFI = .975, GFI = .951, RMSEA = .057, SRMR = .0355. Fig 2 presents the standardized path coefficients. According to the findings, academic self-concept significantly and positively predicted learning engagement (*β* = .514, *p* < .001), and significantly and negatively predicted academic procrastination (*β* = -.251, *p* < .001) within EFL blended learning context. Furthermore, course experience significantly and positively predicted learning engagement (*β* = .449, *p* < .001), and significantly and negatively predicted academic procrastination (*β* = -.223, *p* < .001) within EFL blended learning context. In addition, academic procrastination significantly and negatively predicted learning achievement (*β* = -.124, *p* < .001), while learning engagement significantly and positively predicted learning achievement within EFL blended learning context (*β* = .256, *p* < .001).

### Mediational analyses

The bootstrapping method in SEM was further used to analyze the mediating effect of learning engagement and academic procrastination (the sampling frequency was 2,000). According to Shrout and Bolger, when the 95% confidence interval (CI) of the indirect effect does not contain zero, the mediation effect is significant [130]. In addition, when the indirect effect is significant and the 95% confidence interval (CI) of the direct effect does not contain zero, the mediation effect is a partial mediation effect. On the contrary, the mediation effect in this study is a full one.

From Table 2 and Fig 2, the results showed that on the path from academic self-concept to learning achievement, the direct effect value was .677 and the mediating effect value was .405 (.077 + .328). More specifically, for the mediating effect produced by path one (academic self-concept → learning engagement → learning achievement), the indirect effect was. 328, and for path two (academic self-concept → academic procrastination → learning achievement), the indirect effect was .077. The results showed that the bias-corrected 95% and percentile 95% CI of path one were [.165, .527] and [.152, .501], respectively, indicating that the mediating effect of learning engagement was significant. Thus, the findings supported H2, which held that “the effect of academic self-concept on learning achievement is mediated by learning engagement.” In addition, the bias-corrected 95% CI (.155, .537) and percentile 95% CI (.139, .524) of path two suggested that the mediation effect of academic procrastination was significant. These findings supported H4, which held that “the effect of academic self-concept on learning achievement is mediated by academic procrastination.” More importantly, the bias-correction and percentile 95% CI of the direct effect of the paths were [.417, .922] and [.429, .936], which do not contain zero, indicating that the effect of academic self-concept on learning achievement was partially mediated by learning engagement and academic procrastination.

**Table 2:** Mediating effect test of english learning engagement and english academic procrastination.

Mediating Effect	Point Estimates	Product of Coefficient		Bootstrapping			
				Bias-Corrected 95% CI		Percentile 95% CI	
		SE	Z	Lower	Upper	Lower	Upper
**Indirect Effect 1**: academic self-concept→learning engagement→learning achievement	.328	.090	3.644	.165	.527	.152	.501
**Indirect Effect 2**: academic self-concept→academic procrastination→learning achievement	.077	.030	2.567	.155	.537	.139	.524
**Direct Effect**: academic self-concept→learning achievement	.677	.130	5.208	.417	.922	.429	.936
**Indirect Effect 3**: course experience→learning engagement→learning achievement	.485	.128	3.789	.255	.752	.232	.732
**Indirect Effect 4**: course experience→academic procrastination→learning achievement	.117	.049	2.388	.042	.238	.034	.223
**Direct Effect**: course experience→learning achievement	.286	.231	1.239	-.161	.721	-.152	.730

On the path from course experience to learning achievement, the direct effect value was .286 and the mediating effect value was .602 (.485 + .117). Specifically, for the mediating effect produced by path one (course experience → learning engagement → learning achievement), the indirect effect was .485, and for that produced by path two (academic self-concept → academic procrastination → learning achievement), the indirect effect was .117 (see Table 2). The bias-corrected 95% (.255, .752) and percentile 95% CI (.232, .732) of path one indicated that the mediating effect of learning engagement was significant, thus supporting H3, which held that the effect of course experience on learning achievement is mediated by learning engagement. In addition, the bias-corrected (.042, .238) and percentile 95% CI (.034, .223) of path two suggested that academic procrastination had a significant mediation effect, thus supporting H5, which held that “the effect of course experience on learning achievement is mediated by academic procrastination.” Further, the bias-corrected and percentile 95% CI of the direct effect of the paths were [-.161, .721] and [-.152, .730], which contain zero, indicating that the effect of course experience on learning achievement is fully mediated by learning engagement and academic procrastination (see Table 2).

## Discussion

The present study investigated the relationship and mediation mechanism among university students’ different types of learning preparation (i.e., academic self-concept and course experience), types of learning methods (i.e., learning engagement and academic procrastination), and learning achievement within the Chinese EFL context. With the updates to teaching methods in the post-pandemic era, research has seldom explored the students’ entire EFL learning process and mechanism from the perspective of learners, especially when the 3P teaching and learning model serves as its theoretical basis. This study collected data from a sample of Chinese freshman students who enrolled in an EFL course taught using the blended teaching model. The latent SEM technique was employed to investigate how academic self-concept and course experience relate to the subsequent learning engagement and academic procrastination, thereby influencing the final learning achievement.

However, shaped by the 3P teaching and learning theory, the effect of the mechanism of learning preparation on achievement is complex, meaning that academic self-concept and course experience alone are not enough to guarantee EFL achievement. Therefore, a parallel multiple mediation model was built to clarify whether academic self-concept and course experience influenced EFL learning achievement among the students via the mediators of learning methods—namely, learning engagement and academic procrastination. The findings ascertained that for Chinese university students, the effect of academic self-concept on learning achievement was partially mediated by learning engagement and academic procrastination, and the effect of course experience on learning achievement was fully mediated by learning engagement and academic procrastination. The results of this research prove beneficial for answering the key question about the EFL mechanism and university students’ process of learning in the post-pandemic era, wherein blended teaching models have become widely adopted. This study makes a novel contribution to the literature by illuminating the mediating effect of learning method variables (i.e., academic self-concept and course experience) between learning preparation variables (i.e., learning engagement and academic procrastination) and learning achievement in Chinese university students’ EFL learning within the blended teaching model context. Accordingly, the results suggest that in addition to improving EFL learning preparation, EFL learning engagement must be secured and academic procrastination must be avoided to fully leverage the predictive effect of learning preparation on EFL achievement.

Hypothesis 1a and 1b, which held that academic self-concept and course experience can directly predict learning achievement in Chinese university students’ EFL learning, was supported. Based on the 3P teaching and learning theory, a large number of studies have shown that learning preparation positively correlates with learning achievement [e.g., 58–60,131,132]. The present study contributes to the literature by offering empirical evidence that external learning preparation—that is, course experience—has a predictive effect on learning achievement, and internal learning preparation—that is, academic self-concept—also predicts learning achievement, based on samples of Chinese university students’ EFL learning within the blended teaching model context.

Hypothesis 2, which held that the effect of academic self-concept on learning achievement is mediated by learning engagement, and hypothesis 4, which held that the effect of academic self-concept on learning achievement is mediated by academic procrastination, were also supported. From the results of this research, we can see that for Chinese university students within an EFL blended teaching context, academic self-concept not only can directly predict learning achievement but can also indirectly predict learning achievement through two different learning methods: learning engagement and academic procrastination. According to the self-determination theory, students can make free choices regarding their learning actions based on a full understanding of their needs [133]. Therefore, the higher the academic self-concept of EFL university students, the more objective and optimistic their evaluation of their own learning ability and needs will be. In this way, they may develop the optimistic view that learning achievement is determined by one’s internal qualities rather than external factors, leading them to take a more active role in learning English and benefit from a high level of learning achievement. Meanwhile, it is difficult for university students who have a lower level of self-concept to develop a positive self-evaluation. These students often fail to deal with problems with a proactive and objective attitude, which has an adverse effect on their choice of follow-up learning methods. As a result, they engage in academic procrastination in EFL courses, which in turn negatively influences their learning achievement (i.e., final EFL course scores).

Hypothesis 3, which held that the effect of course experience on learning achievement is mediated by learning engagement, and hypothesis 5, which held that the effect of course experience on learning achievement is mediated by academic procrastination, were also supported. In this model, learning methods served as full mediators between learning preparation and learning achievement, indicating that course experience predicted learning achievement of university students through learning engagement as well as academic procrastination, but that course experience alone cannot predict or explain the variation of learning achievement. Thus, it can be seen that within the blended teaching model context, teachers’ rich teaching content, diversified teaching approaches, and flexible teaching methods cannot improve EFL learning achievement of university students without the presence of additional elements. Good academic achievement within the blended teaching model potentially results from the fact that this model focuses on students’ experience in the learning process rather than learning achievement [134]. The blended teaching model centers on impelling students to transform from “passive learners” to “active learners.” This teaching method, which prioritizes the development of students’ autonomous learning ability, pays much more attention to the organic combination of language learning and ability-building than the summative quantitative evaluation index [135,136]. Therefore, course experience has a closer relationship with learning methods within this model but cannot directly predict the final EFL examination scores.

The present research reveals that both students’ academic self-concept and course experience affect learning engagement within the EFL blended teaching context. This reflects that both individual learning preparation and external teaching preparation have close connections with learning engagement. Learning engagement of Chinese university students is essentially an outcome of the interaction between individual factors and the external environment. This finding aligns with the concept of triadic reciprocal determinism, which holds that individuals’ behavior is shaped by the interconnection and interaction between personal characteristics, environment, and behavior [137,138]. It can also be explained by the reciprocal causation model proposed by Bandura. According to Bandura’s model, an individual’s behavior both influences and is influenced by personal factors and the environment [139]. Within the EFL blended learning context, a student’s decision of whether to invest in learning or to procrastinate is influenced by personal factors such as self-concept as well as environmental elements such as course factors.

It should be noted that compared to external teaching preparation, individual learning preparation is more closely associated with different types of learning methods and the final learning achievement. Thus, although both individual factors and external environmental factors can have a direct effect on learning engagement of university students, individual factors have a stronger predictive power compared to the external environmental factors, and students’ decision of whether to choose positive or negative learning methods in an online environment is more strongly influenced by personal factors than external factors, especially teaching-related factors—which have long been considered the most important influences on learning within the traditional classroom setting. This finding highlights the important role that individual learning preparation plays in the EFL learning process within the blended teaching model.

### Teaching suggestions and countermeasures

Based on the 3P teaching and learning model, the present study discussed learning process and mechanism of university students within an EFL blended teaching model context. Below are some suggestions and countermeasures for effectively applying this model in EFL teaching situation in China’ s colleges and universities and improving the effect of university students’ EFL learning in two aspects, i.e., individual learning preparation and external teaching preparation.

Firstly, here is a need to improve academic self-concept in EFL to increase individual preparation for study. The present study found that individual learning preparation is one of the most effective factors for improving EFL learning engagement and learning achievement, in which the establishment of individual academic self-concept is of vital importance. In view of this, teachers should not only provide students with analysis of one’s own advantages in EFL learning and thus improve their learning confidence, but also practice the “cooperativity” principle in blended teaching and encourage students to carry out competitions between groups in a bid to enhance their EFL learning competency. Moreover, students should take proactive measures to improve their academic self-concept and learning methods, for example, avoid shying away from learning EFL, change learning strategies by measuring their own strengths and shortcomings, improve their learning regulation ability, spontaneously set up a learning warning system to avoid sidestepping learning tasks, thereby eventually achieving the autonomy and persistence of EFL learning.

Secondly, the EFL course needs to be optimized to increase the external teaching preparation. The present study found that external teaching preparation, which have converted to students’course experience to investigate, is one of the assisting means of improving students’ EFL learning methods and achievement. On the one hand, teachers should be equipped with rich experience in blended teaching, especially the ability to bring smart technology into teaching and achieve higher-level integration of information technology and classroom teaching. Teachers also need to expand theoretical knowledge about blended teaching and subject teaching approaches, set clear and effective teaching objectives and standards, improve the skills to efficiently integrate resources and organize activities through diversified channels, adopt a two-way model of formative evaluation and summative assessment, pay attention to students’ feelings to assess the classroom instruction, etc. On the other hand, universities should lay down sound policies for the reform of blended teaching, make clear and strategic plans for the blended teaching, create a favorable platform environment and support systems, and provide mature models or process frameworks for the blended teaching of EFL, so as to set the stage for blended teaching practices of the teachers and guarantee successful and high-quality blended teaching in EFL classrooms.

## Conclusion

This study aimed to investigate the association between learning preparation and learning achievement and the mediation mechanism between these two constructs in a sample of freshman students within a blended teaching model context. The findings showed that students’ different aspects of learning preparations (i.e., academic self-concept and course experience)had direct and indirect predictive effects on learning achievement in Chinese university students’ EFL learning. In addition, the results also indicated that different styles of learning methods(i.e., learning engagement and academic procrastination) played a mediating role between learning preparations and learning achievement. The findings can add new knowledge to the literature on university students’ learning achievement by uncovering how learning preparation may play a role with the mediation of methods. This study can be the first to reveal the association between these variables related to EFL learning of university student by 3P theory perspective. In addition, the results also add empirical evidence that the 3P theory is still applicable to the teaching and learning practice using the emerging blended teaching model in the post-epidemic era.

Several limitations need to be noted. Firstly, samples in the present study were university freshman students, which might be not conducive to generalizing our results to all university learners within the EFL blended teaching context. Samples from various educational background and academic backgrounds and of other university grades should be considered in future exploration. Secondly, we only located two aspects of learning preparation (i.e., academic self-concept and course experience) as the independent variables and two types of learning methods (learning engagement and academic procrastination) as the mediators. However, based on the 3P theory, the preparation variables in presage phase also include other learning quality such as expectations, and classroom climate and the preparation variables in process phase also include learning strategies such as deep learning or rote. As frequently examined in the 3P teaching and learning model-related empirical literature, more preparation variables may join force with relevant preparation variables above to influence learning achievement [140–143]. Future research needs to incorporate more variables to examine the adaptation of 3P theory model in the environment of EFL blended teaching. Third, the present study is a cross-sectional examination, which limits causal and directional hypotheses. Freshman students’ learning achievement may be more strongly affected by previous learning methods in high school or prior EFL-related self-concept could not be tracked. Their experience of EFL courses, their self-concept of English and their approach to learning EFL may change over time. Even the 3P teaching theory has discussed that previous learning achievement will further change the preparation for later learning, and thus have a new impact on the future learning process [38]. It is therefore meaningful to conduct longitudinal studies to further examine the dynamics of the relationship between learning preparation, learning methods and learning achievement, so as to strengthen the argument for the causality of them.

## Supporting information

S1 Dataset (SAV)

S1 File (AMW)
